# Spatial Variations of Heavy Metals in the Soils of Vegetable-Growing Land along Urban-Rural Gradient of Nanjing, China

**DOI:** 10.3390/ijerph8061805

**Published:** 2011-05-25

**Authors:** Shi-Bo Fang, Hao Hu, Wan-Chun Sun, Jian-Jun Pan

**Affiliations:** 1 Chinese Academy of Meteorological Sciences, Zhongguancun South Street 46, Beijing,100081, China; E-Mail: sbfang0110@163.com; 2 Zhejiang Academy of Agricultural Sciences, Shiqiao Road 198, Hangzhou, 310021, Zhejiang Province, China; E-Mails: huhao82@126.com (H.H.); sunwc76@yahoo.com.cn (W.-C.S.); 3 Nanjing Agricultural University, No.1 Weigang, Nanjing, 210095, Jiangsu Province, China

**Keywords:** urbanization, heavy metal, soil, spatio-temporal distribution

## Abstract

China has experienced rapid urbanization in recent years. The acceleration of urbanization has created wealth and opportunity as well as intensified ecological and environmental problems, especially soil pollution. Our study concentrated on the variation of heavy metal content due to urbanization in the vegetable-growing soil. Laws and other causes of the spatial-temporal variation in heavy metal content of vegetable-growing soils were analyzed for the period of urbanization in Nanjing (the capital of Jiangsu province in China). The levels of Cu, Zn, Pb, Cd and Hg in samples of vegetable-growing soil were detected. The transverse, vertical spatio-temporal variation of heavy metals in soil was analyzed on the base of field investigations and laboratory analysis. The results show that: (1) in soil used for vegetable production, the levels of heavy metals decreased gradually from urban to rural areas; the levels of the main heavy metals in urban areas are significantly higher than suburban and rural areas; (2) the means of the levels of heavy metals, calculated by subtracting the sublayer (15–30 cm) from the toplayer (0–15 cm), are all above zero and large in absolute value in urban areas, but in suburban and rural areas, the means are all above or below zero and small in absolute value. The causes of spatial and temporal variation were analyzed as follows: one cause was associated with mellowness of the soil and the length of time the soil had been used for vegetable production; the other cause was associated with population density and industrial intensity decreasing along the urban to rural gradient (*i.e.*, urbanization levels can explain the distribution of heavy metals in soil to some extent). Land uses should be planned on the basis of heavy metal pollution in soil, especially in urban and suburban regions. Heavily polluted soils have to be expected from food production. Further investigation should be done to determine whether and what kind of agricultural production could be established near urban centers.

## 1. Introduction

Urbanization, can be viewed as a typical phenomenon of economic growth and industrial advancement. Due to the increasing rate of urbanization, there continues to be concern about the impact of anthropogenic activities on urban and suburban soil. Urbanization and industrialization have changed the urban space into specific urban ecosystems and the soil is an important component of these systems [1]. The original structure and properties of soil have been deeply modified, and new soils with particular characteristics, the anthroposols, have been created [2]. Recently, much research has been done on urban and industrial soils including: (1) the studies on spatio-temporal distribution of heavy metals and their functional roles along industrialization gradients within single cities [3–6]; (2) the research concentrated on different possible sources for the enrichment of heavy metals in soils [7–10]; and (3) due to bioavailability and toxicity of heavy metal in soil in food chains via plant uptake, the studies on human health risks and control measures of soil heavy metal pollution [11–16]. However, these studies focused mainly on limited locations, particularly in urban areas or major pollution sources. In contrast, there are few extensive surveys on heavy metal distributions in soils along an urban-rural gradient, especially in agricultural soils.

The average urbanization level in China was approximately 12% in 1950, 29% in 1996, 32% in 1999, and 36% in 2000 [17]. Excessive accumulation of heavy metals in soils, especially in agricultural soils may not only result in environmental contamination, but elevated heavy metal uptake by crops may also affect food quality and safety. Owing to rapid economic development, heavy metal contamination of urban and peri-urban agricultural soils has also become increasingly serious in China [18–21]. Urban and peri-urban agricultural soils can contribute substantial amounts to the proportion of food consumed in the city, for example, it estimated that 15–20% of the vegetables and meat is produced on peri-urban agricultural soils in Nanjing City. This potential risk indicates that there is an urgent need to conduct further studies into heavy metal contamination of urban and peri-urban agricultural soils. Most of soil heavy metals come from industry resources or agricultural resources or the sediments. However, there were few reports to analyze which were the mainly pollution sources of heavy metals in vegetable-growing soil near urban.

The present investigation aims to: (1) investigate the distribution of heavy metals (Pb, Zn, Cr, Hg and Cu) in vegetable plot soil using urban–rural gradients in Nanjing city, (2) compare some current data with those of a 1985 sampling, and (3) analyze the possible pollutant sources which could cause the accumulation of soil heavy metals in vegetable plots.

## 2. Materials and Methods

### 2.1. Study Area

Nanjing region, with a population of 5.63 million and covering 6,597 km^2^, lies in eastern China (31°14′–32°36′ N, 118°22′–119°14′ E). It is in the process of rapid economic growth, urbanization and industrialization. About 15.6% of this region is urbanized.

### 2.2. Soil Samples Collection and Preparation

**Figure 1.**
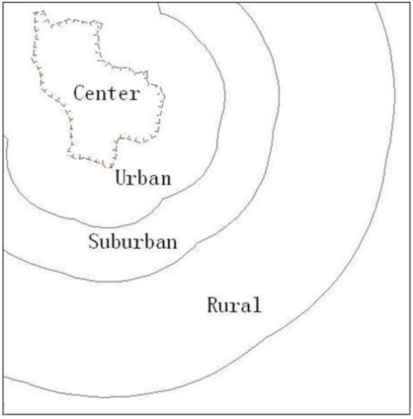
Map of urban, suburban and rural regions distribution.

Fourteen vegetable plots were sampled from three concentric rings around the city of Nanjing. The centre-most, urban ring extends from the city wall (which surrounds the centre of the city) to a distance of 4.0 km and encompasses 5 of the plots. Next, 5 sites were sampled from the suburban ring which extends from 4.0 km to 7.5 km. Finally, 4 rural sites were sampled from the outer-most ring extending 7.5 to 15 km from the city wall (Figure 1). All the sample sites are far away from the point pollution sources (such as landfill regions, gas station and factories) and line pollution sources (about 200 meters away from rivers and roads). All sites were located with GPS in order for further investigation. Investigation results of the sample plots’ surrounding environment, such as geographical and geological conditions, hydrological distributions, and neighboring pollution sources, were recorded. The soil managing conditions including the cultivation time, fertilization frequencies and quantities were also investigated.

### 2.3. Sample Treatment and Data Analysis

Each soil sample consists of 35 sub-samples random collected from the sampling plot of about 200 m × 200 m. At each sampling point, the top-layer sample (0–15 cm of the soil) and sub-layer sample (15–30 cm) was taken separately using a stainless steel sampling tube. The soil samples were then placed into polyethylene bags, and returned to the laboratory. All these samples were air dried at room temperature and sieved through a 0.850 mm nylon sieve to remove coarse debris. The soil samples were then ground with a agate pestle and mortar until all particles passed a 0.150 mm nylon sieve. For the total heavy metal content analysis, Total Zn, Pb and Cr contents in soils were determined by an Atomic Absorption Spectrophotometer after digesting in HNO_3_–HF–HClO_4_. The total Cu contents in soils were determined by the Atomic Absorption Spectrophotometer after digesting in HNO_3_–HCl–HClO_4_ mixture. Total Hg contents in soils were digested in H_2_SO_4_–KMnO_4_ mixture and determined by a Cold Atomic Absorption Spectrometer (CAAS).

Pearson’s correlation coefficients and Principle Component analysis (PCA) of heavy metal elements in top-layer and sub-layer were calculated and one-way ANOVA method to test the significant differences of heavy metals content in urban, suburban and rural regions was employed by SPSS software for Windows, version 13.0 (SPSS Inc., USA).

## 3. Results and Discussion

### 3.1. Top-layer Heavy Metals Variation Along the Urban-Rural Gradient

The results showed that the top-layer (0–15 cm) heavy metal levels in the soil varied greatly in urban (0–4 km), suburban (4–7.5 km) and rural regions (7.5–15 km) (Table 1). Except for Zn, the mean values of all heavy metals were degressive from urban to rural environment. All heavy metal contents, including Zn, were higher in urban sites than those in suburban and rural sites. Analysis of variance (ANOVA, *p* < 0.05) showed that there were significant differences in heavy metals between the urban and suburban areas for Pb, Zn, Hg, and Cu, whereas no significant difference was found between suburban and rural region. There were no significant differences among the 3 types of regions for Cr.

### 3.2. Vertical (Soil Profile) Variation of Heavy Metals along the Urban-Rural Gradient

Many researchers concluded that heavy metal pollution in soil is mainly concentrated in the upper layer (0–10 cm) of the top ploughed layer (0–15 cm) of the artificial cultivated soil profile [22–24]. Therefore, comparing the heavy metal contents in top-layer and sub-layer soils show the heavy metal distribution in the soil profiles. The mean levels were calculated by subtracting the sub-layer (15–30 cm) from the top-layer (0–15 cm). According the subtracted results, Significance tests (ANOVA in probability levels of <0.05) were done and the results are shown in Table 2. The mean contents of heavy metals, which were calculated by the top-layer (0–15 cm) subtracting the sub-layer (15–30 cm), are all above zero and large in absolute value in urban areas, but in suburban and rural areas the means are all near zero and small in absolute value. There are no significant differences between different regions.

### 3.3. Identification of the Potential Pollutant Sources which Accumulate Heavy Metals

This research suggests that almost all investigated heavy metals accumulated in the top-layer in vegetable soil in urban areas where, as we know, there is relatively more industrial activity and more automobile emission sources than in suburban and rural areas. Variations of heavy metals along the urban-rural gradient in soil profiles indicate a distribution pattern in urban areas unaffected by regional background sources, mainly controlled by local sources.

Industrialization and urbanization as major sources of heavy metal pollution are known by many authors [25,26]. Nanjing is one of the fastest economic growth areas and is in the process of rapid urbanization and industrialization [27]. Generally, the proximity of vegetable plot soil to urban areas could increase pollution from irrigation with polluted waters, fertilization with contaminated manure, atmospheric particle fall from industrial dust, combustion of fossil fuels and road traffic. But which were the main pollution sources?

#### 3.3.1. Pollution source analysis

According to further analyses, we find that urban heavy metal accumulation in soil depends on how long the vegetable-growing soil is cultivated. As Table 3 shows, the cultivation time for most urban sample plots was much longer than suburban and rural plots. The literature also indicates that top-layer heavy metals are increasing evident as the cultivation time increases [28,29]. It suggests that urban heavy metals in vegetable-growing soil could be caused by long-time vegetable cultivation and corresponding operation. Contaminated manure, water irrigation and intensifying use of fertilizers are the main pollution sources of heavy metal in soil [30]. But in all the agro-measurement, which are major pollution sources?

Another research report can give some indications of major pollution sources in Nanjing [20]. The study area was located 5 km east of Nanjing. Compare to the places which we had sampled, the places which they had sampled was limited to urban areas, which were comparable to the urban places we had researched. Some of the results serve to explain some of our results, especially for urban sampled soil pollutions in our investigation. In their paper, the concentrations of heavy metals in irrigation water, chemical fertilizer, and organic wastes (cow manure) were investigated. It found that the concentrations of all heavy metals measured in irrigation water samples in Nanjing were much lower than the most stringent grade of the Chinese environmental quality standards for surface water—National standard I [31]. It also found high concentrations of heavy metals such as Cu and Zn in cow manure, but none of the chemical fertilizers contained high concentrations of heavy metals. All these indicated that much of soils heavy metals in vegetable-growing land originated from heavy applications of cow manure.

Could the soils were accumulating heavy metal from industrial, transportation, atmospheric sedimentation and the other sources? Suspended particulates (TSP) in the atmosphere of Nanjing compared with the background values of soil indicated that atmospheric dustfalls have elevated heavy metal concentrations as a whole, except those of Cr which mainly derived from soil particles [32]. Pb (46%–64% in total)and Cu (49%–73% in total) were residual fraction, whereas Zn (31%–69% in total) mainly in the oxidizable fraction in soils in suburban of Nanjing [33],which indicate that majority Zn was mainly from artificial contaminant, a majority of Cu and Pb were mainly from soil parent material. Another research implied that Pb mainly accumulates from heavy traffic, Cu mainly accumulates from cow manure, and Zn accumulate by irrigation with sewage and urban surface water [34]. All these suggested that different metal varied in main contaminant sources.

#### 3.3.2. Correlation coefficient analysis in heavy metals

It has been confirmed in our research that the concentrations of most soil heavy metals are significantly higher in urban than suburban and rural areas. And there were no significant differences between suburban and rural regions (Table 1). Therefore, Pearson’s correlation coefficients of heavy metal elements in top-layer and sub-layer were calculated in two groups (1) urban and (2) suburban and rural regions. They are summarized separately in Tables 4, and 5.

From Table 4, in the top-layer of urban soil, Pb, Zn Cu, and Hg are significantly positively correlated, which may suggest a common origin, while Cr is negatively correlated with the other metals, reflecting different sources of Cr from other elements. No significant differences were found among the 3 types of regions (urban, suburban and rural) for Cr (Table 1), which indicated that no or little artificial contaminant accumulated Cr. All these suggested that Cr probably derived from soil parent material in urban soil. In the top-layer of the suburban and rural regions, there are no significant correlations among Pb, Zn Cu, and Hg, while there is a significant positive correlation between Cr and Cu. From Table 5, in the sub-layer of urban soil, Pb and Hg are significantly positively correlated, while in the suburban and rural regions, Pb are significantly positively correlated with Zn and Cr. Many researchers indicated that heavy metal pollution in soil is mainly concentrated in the upper layer (0–10 cm) of the top ploughed layer [22–24]. Correlation coefficient of sub-layer of our research induced that Pb, Zn and Cr were likely from soil parent material in suburban and rural regions.

#### 3.3.3. Principle Component Analysis (PCA) in top-layer and sub-layer

**Figure 2.**
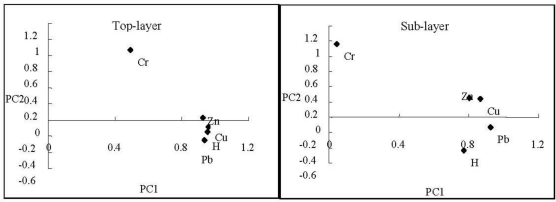
PCA loading 2-D plots (PC1 *vs*. PC2) for 5 heavy metals.

PCA and cluster analysis (CA) are the most common multivariate statistical methods used in environmental studies [35,36]. In order to identify the heavy metals relationship, heavy metal elements in top-ayer and sub-layer were calculated separately by PCA method. 2-D plots of the PCA loadings in top-lay and sub-layer are presented in Figure 2. (Because two factors can account for 92.93% of the total variance in top-layer, and 81.93% in the sub-layer, 2-D plots (PC1 *vs*. PC2) of the PCA were draw.)

The relationships among the five heavy metals are readily seen in Figure 2. In the top-layer plot, Cr and a group (Zn, Cu, Hg and Pb) are separated by a large distance in the 2-D PCA loading plot, which may suggest that the two are poorly correlated and have different sources. In the sub-layer plot, Cr, Pb, Hg and a group (Zn, Cu) are separated clearly in the 2-D PCA loading plot, which may suggest they have different sources.

### 3.4. Land-Use Planning Concerned with Heavy Metal Pollution

As the above analyses show, almost all heavy metals’ mean are degressive along with the urban to rural gradient, and vegetable soils were contaminated with Zn, Pb, Hg and Cu, especially in urban region [5]. Urban farmland soil pollution with heavy metal has been reported in many cities in the world. Heavy metals can exert detrimental effects on human health and on the environment in big cities [26]. Pb, Zn, Cr, Cd and Hg, which are considered very toxic elements, are of primary concern in soil and food contamination in recent literature [11,37–39]. Data had demonstrated that both Pb, Cd and Hg in vegetables posed substantial risk to local residents in China [40,41]. Heavy metals, such as Pb and Zn from heavy traffic, Hg from thermoelectricity power stations and combustion of fossil fuels, accumulated in most big cities in China. However, in the current study, regions near to urban and the suburban primarily plant vegetable and are the main vegetable production areas of cities, because that planting vegetables in urban and the suburban is more convenient transportation and better economic benefit than rural regions. Metal concentrations in vegetables and vegetable consumption through the foodchain poses a substantial risk to local residents, although the actual risks to local populations remains to be examined. The procedures of soil remediation are expensive and sometimes difficult to perform. Therefore heavily polluted soils have to be excepted from food production [42] and land use planning should be based on heavy metal pollution in the soil. Generally, we should avoid production of leafy vegetable and root plants in polluted areas, whereas leguminous plants and fruit plants could be grown under certain conditions [43]. Further investigation should be done to determine whether and what kind of agricultural production could be established in areas in close proximity to urban areas. Heavily polluted areas, such as urban regions, should avoid food production according to relevant reference standards and should be altered to non-agricultural land-use (recreation areas, construction sites, trade and industry areas, *etc.*).

## 4. Conclusions

The conclusions show that: (1) In soil used for vegetable production, the contents of heavy metals decrease gradually from urban to rural areas; the contents of main heavy metals in urban areas are significantly higher than suburban and rural areas; (2) the means of the levels of heavy metals, which were calculated by subtracting the sub-layer (15–30 cm) from the top-layer (0–15 cm), are all above zero and large in absolute value in urban areas, but in suburban and rural areas, the means are all near zero and small in absolute value. The findings presented here indicate that the location and the time frame of cultivation are important factors in determining the extent of heavy metal levels. Almost all heavy metals’ means are degressive along the urban-rural gradient, and vegetable soils were severely contaminated by Zn, Pb, Hg and Cu, especially in urban regions. Land-use planning should be considered as a good choice to avoid vegetable consumption posing substantial risk to local residents.

## Figures and Tables

**Table 1.** One-way ANOVA of top-layer soil heavy metal contents.

**Heavy metals**	**Location**	**N**	**Mean**	**S. D.**	**Range**
Pb (mg/kg)	Urban	5	65.23 ^a,*^	17.46	45.95–83.54
	Suburban	5	36.61 ^b^	1.16	35.07–38.14
	Rural	4	32.10 ^b^	5.98	26.70–38.68

Zn (mg/kg)	Urban	5	224.75 ^a^	36.35	191.78–282.54
	Suburban	5	122.11 ^b^	20.14	99.67–150.07
	Rural	4	144.69 ^b^	46.80	81.40–191.66

Cr (mg/kg)	Urban	5	67.49	7.50	58.22–74.88
	Suburban	5	60.09	13.07	43.58–77.42
	Rural	4	53.98	14.10	40.10–68.71

Hg (mg/kg)	Urban	5	0.494 ^a^	0.146	0.281–0.622
	Suburban	5	0.176 ^b^	0.040	0.122–0.233
	Rural	4	0.136 ^b^	0.058	0.094–0.224

Cu (mg/kg)	Urban	5	50.17 ^a^	13.43	38.67–72.40
	Suburban	5	29.27 ^b^	4.94	23.35–35.35
	Rural	4	24.93 ^b^	4.89	20.20–31.38

* ^a^ and ^b^ denote they have statistical significance at probability levels of <0.05; ^a^ and ^a^ or ^b^ and ^b^ denote they have no statistical significance.

**Table 2.** Significance test (ANOVA) of subtracted results along with the gradient.

**Heavy metal**	Urban(n* = 5)	**Suburban (n = 5)**	**Rural (n = 4)**
Pb (mg/kg)	13.05	1.91	−1.74
Zn (mg/kg)	55.44	−2.72	11.1
Cr (mg/kg)	15.06	−1.75	−3.1
Hg (mg/kg)	0.087	−0.041	0.051
Cu (mg/kg)	6.68	−0.61	−4.39

* n is the number of the sample.

**Table 3.** Vegetable farming period in different area.

	**Urban**					**Suburban**					**Rural**			
Site no.	1	3	4	5	6	2	8	9	10	12	7	11	13	14
Cultivated time (year)	20	20	40–50	10–20	20–30	2	20–30	2	3	10	5–6	10	5–6	2

**Table 4.** Pearson’s correlation matrix for the metal concentrations of top- layer of soil.

	**Urban**				**Suburban and rural area**			
**Element**	**Pb**	**Zn**	**Cr**	**Hg**	**Pb**	**Zn**	**Cr**	**Hg**
Pb								
Zn	0.843 *				0.045			
Cr	−0.767 *	−0.397			0.634	0.543		
Hg	0.874 *	0.892 **	−0.606		0.240	0.363	0.318	
Cu	0.888 **	0.939 **	−0.608	0.857 *	0.570	0.124	0.818 *	0.209

The left lower part is the correlation coefficient; the right upper part is the significance level.

* P < 0.05 (2-tailed);

** P <0.01 (2-tailed).

**Table 5.** Pearson’s correlation matrix for the metal concentrations of sub-layer of soil.

	**Urban**				**Suburban and rural area**			
**Element**	**Pb**	**Zn**	**Cr**	**Hg**	**Pb**	**Zn**	**Cr**	**Hg**
Pb								
Zn	0.202				0.772 *			
Cr	−0.590	0.011			0.885 **	0.553		
Hg	0.903 **	0.057	−0.416		−0.176	0.026	−0.322	
Cu	0.422	0.307	0.298	0.278	0.723	0.594	0.468	−0.010

The left lower part is the correlation coefficient; the right upper part is the significance level.

* P < 0.05 (2-tailed);

** P <0.01 (2-tailed).
